# Influence of Simulated Wound Exudate on the Antimicrobial Efficacy of Various Intracanal Medicaments Against Enterococcus faecalis: An In Vitro Study

**DOI:** 10.7759/cureus.38677

**Published:** 2023-05-07

**Authors:** Ilango Sangita, Sankar Vishwanath, Kadandale Sadasiva, Anupama Ramachandran, Yashini Thanikachalam, Vengidesh Ramya

**Affiliations:** 1 Conservative Dentistry and Endodontics, Chettinad Dental College and Research Institute, Chennai, IND; 2 Conservative Dentistry and Endodontics, KSR (K.S. Rangasamy) Institute of Dental Science and Research, Erode, IND

**Keywords:** periapical exudate, enterococcus faecalis, bovine serum albumin, cetylpyridinium chloride, intracanal medicament

## Abstract

Aim

The aim of this study is to compare and evaluate the antimicrobial efficacy of chlorhexidine, calcium hydroxide, and cetylpyridinium chloride against *Enterococcus faecalis* in the presence and absence of contamination with simulated periapical exudate at different time intervals.

Methods

Simulated wound exudate and cetylpyridinium chloride gel were prepared prior to testing. The test groups were divided into groups A and B based on the presence and absence of simulated wound exudate. They were further divided into four subgroups as follows: subgroup 1: calcium hydroxide; subgroup 2: 2% chlorhexidine gel; subgroup 3: 0.5% cetylpyridinium chloride gel; subgroup 4: 0.9% saline as control. *E. faecalis* was inoculated, and the test groups were evaluated at different time periods of six, 12, and 24 hours. Aliquots were then obtained and subjected to 10-fold serial dilutions. A total of 10 µl of individual samples was spread onto the nutrient agar medium using L-rod. The plates were then assessed for colony-forming units (CFU), and the values obtained were subjected to statistical analysis. Kolmogorov-Smirnov and Shapiro-Wilk normality tests were used to check whether the variables follow a normal distribution. For within-group comparison, the Friedman test and the Kruskal-Wallis test were used. For between-group comparison, the Mann-Whitney U test was used.

Results

Saline had the highest CFU values, while cetylpyridinium chloride had the lowest CFU values in both contaminated and non-contaminated groups. In all the conditions, the CFU values of cetylpyridinium chloride were significantly lowest compared to the other three groups. CFU values of the calcium hydroxide group were significantly high, followed by the chlorhexidine group when compared to cetylpyridinium chloride in both contaminated and non-contaminated groups.

Conclusion

Within the limitations of the current study, it can be concluded that cetylpyridinium chloride was the most effective intracanal medicament against *E. faecalis* than calcium hydroxide and chlorhexidine at varying time intervals, even in the presence of a periapical exudate. Thus, cetylpyridinium chloride can be considered an effective intracanal medicament for root canal disinfection.

## Introduction

Endodontic treatment aims at the elimination of microorganisms and residual pulp tissue from the root canals and helps achieve a proper three-dimensional hermetic seal. However, the presence of various anatomical complexities and aberrations of the root canal systems have rendered mechanical instrumentation insufficient for its complete disinfection [1]. Hence, the need for irrigants and intracanal medicaments comes into play for complete bacterial eradication from root canals.

Intracanal medicaments are introduced into the root canals for the purpose of inhibiting bacterial growth. An ideal intracanal medicament should possess properties such as non-irritating, low surface tension, non-staining, sustained antimicrobial effect, and active in the presence of protein derivatives of pulpal or periapical tissues, serum, and blood [1]. No single intracanal medicament has all the necessary requisites. There exists a continuous demand for an ideal intracanal medicament.

Cetylpyridinium chloride (CPC) is a recently introduced quaternary ammonium compound with unique positively charged surfactant properties. CPC has a negative effect on salivary plaque bacteria and inhibits their ability to form biofilms and is thus used in mouthwashes [2]. Recently, it is gaining popularity due to its incorporation into endodontic irrigants and root canal sealers [3].

In cases of chronic apical periodontitis, the presence of inflammation is noted in the periapical region that can be detected using various immunological markers. The presence of inflammation may be accompanied by the occurrence of periapical exudate. Periapical exudate is an inflammatory exudate that contains locally produced factors like prostaglandin E2 (PGE2), neutrophil elastase, and matrix metalloproteinases (MMPs). It is often present in infected root canals and provides insight to understand the host response in periapical pathoses by evaluating its biochemistry [4].

Endodontic mishaps such as over-instrumentation of root canals during chemo-mechanical preparation can occur in many instances. During such cases, when the intracanal medicament is placed as an inter-appointment dressing, an interaction can occur between the periapical exudate and intracanal medicament at the apical region due to seepage of periapical tissue fluid/exudate into the canal space [5]. To our knowledge, no in vitro studies have been conducted to assess the type of interaction that occurs between periapical exudate and the intracanal medicament.

The aim of this study is to compare and evaluate the antimicrobial efficacy of chlorhexidine (CHX), calcium hydroxide (CH), and CPC against *Enterococcus faecalis* in the presence and absence of contamination with simulated periapical exudate at different time intervals.

The null hypothesis was that there is no significant difference in the antimicrobial efficacy among the various intracanal medicaments used against *E. faecalis* in the presence or absence of simulated periapical exudate.

## Materials and methods

Preparation of simulated periapical wound exudate

The preparation of the simulated periapical exudate was adapted from the study by Bradford et al. (2009) [6]. The components used have been listed in Table 1.

**Table 1:** Composition of simulated wound exudate

Simulated wound exudate
5.8440 g sodium chloride
3.3604 g sodium hydrogen carbonate
0.2982 g potassium chloride
0.2775 g calcium chloride
33.00 g bovine albumin
1,000 mL deionized water

Preparation of cetylpyridinium chloride gel

Calcium chloride was dissolved in distilled water and diluted with 80% of the required amount of N-methyl-2-pyrrolidone (NMP). CPC was added to the solution using a vortex mixer and vigorously stirred to obtain the gel. The solution was left standing at 40°C for another 15 minutes until complete dissolution and stored at room temperature until use.

Microbial cultivation

Pure cell line cultures of *Enterococcus faecalis* (ATCC 29212) were obtained from National Chemical Laboratory, Pune, India. The bacterial strain was diluted in brain heart infusion broth and kept at 37°C for 24 hours in an incubator.

Microbiological testing

The test groups were divided into groups A and B based on the presence and absence of simulated wound exudate. They were further divided into four subgroups based on the medicament used, as seen in Table 2.

**Table 2:** Medicaments tested against Enterococcus faecalis

Subgroups
Subgroup 1: Calcium hydroxide (CH) (RC Cal, Prime Dental Products, Thane, India)
Subgroup 2: 2% chlorhexidine gel (CHX) (Prevest DenPro, Jammu, India)
Subgroup 3: 0.5% cetylpyridinium chloride gel (ALG Chemicals, Sheltoli, India)
Subgroup 4: 0.9% saline as control

In Group A, 1 ml of the medicament test groups was incubated with 1 ml of the simulated wound exudate in sealed test tubes at 37°C for one hour followed by the addition of *Enterococcus faecalis* suspension.

In Group B, 1 ml of the test groups was added to the *Enterococcus faecalis *suspension and gently mixed and incubated at 37°C.

The test groups were evaluated at different time periods of six, 12, and 24 hours after bacterial inoculation. Aliquots were then obtained and subjected to 10-fold serial dilutions. A total of 10 µl of individual samples were spread onto nutrient agar plates using L-rod for 24 hours at 37°C. The plates were inspected under a stereo-microscope for assessment of colony-forming units (CFU). This experiment was done in triplicate. The values obtained were subjected to statistical analysis.

Statistical analysis

Statistical analyses were performed using a personal computer in IBM SPSS Statistics for Windows version 20.0 (IBM Corp., Armonk, NY). Depending upon the nature of the data, the statistical tests were chosen. A p-value of < 0.05 was considered to be significant. Kolmogorov-Smirnov and Shapiro-Wilk normality tests were used to check whether the variables follow a normal distribution. For within-group comparison, the Friedman test and the Kruskal-Wallis test were used. For between-group comparison, the Mann-Whitney U test was used.

## Results

**Figure 1:**
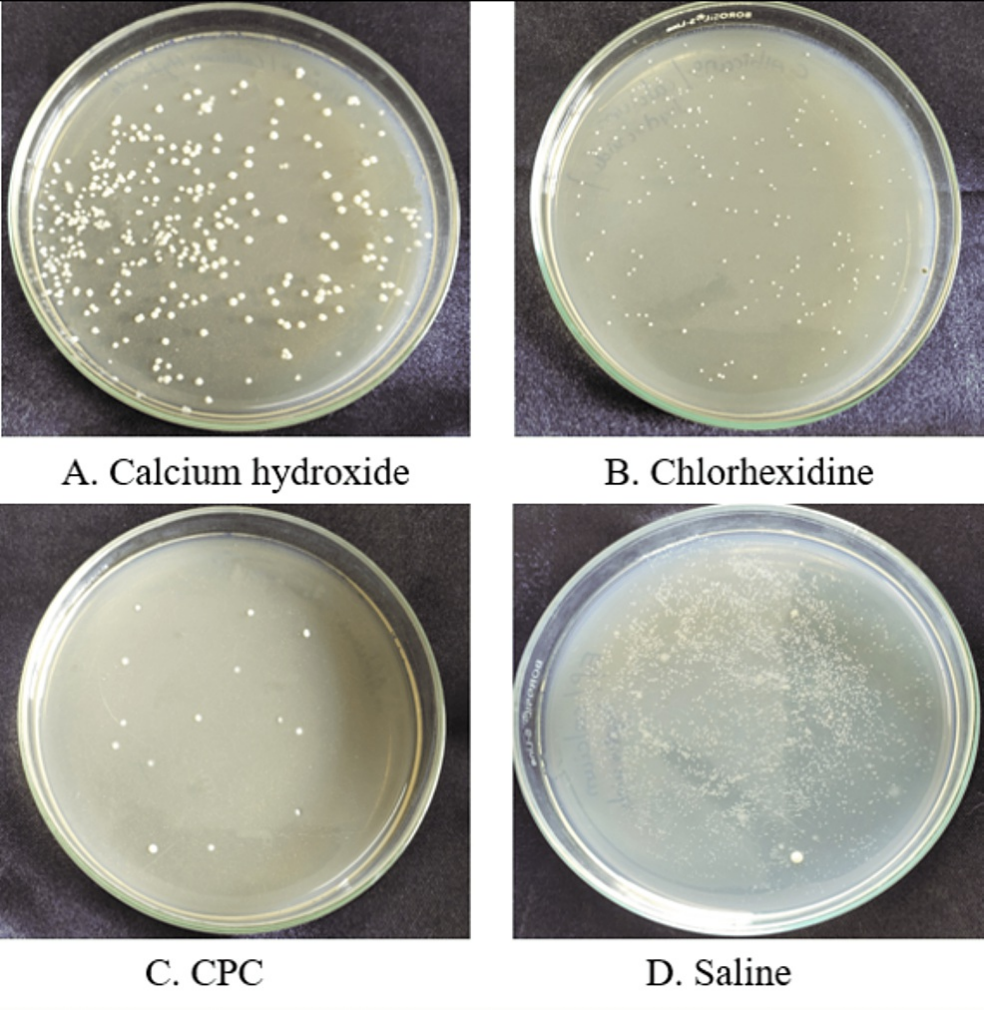
Microbial colony of Enterococcus faecalis of Group A at the 24-hour time interval CPC: cetylpyridinium chloride.

**Figure 2:**
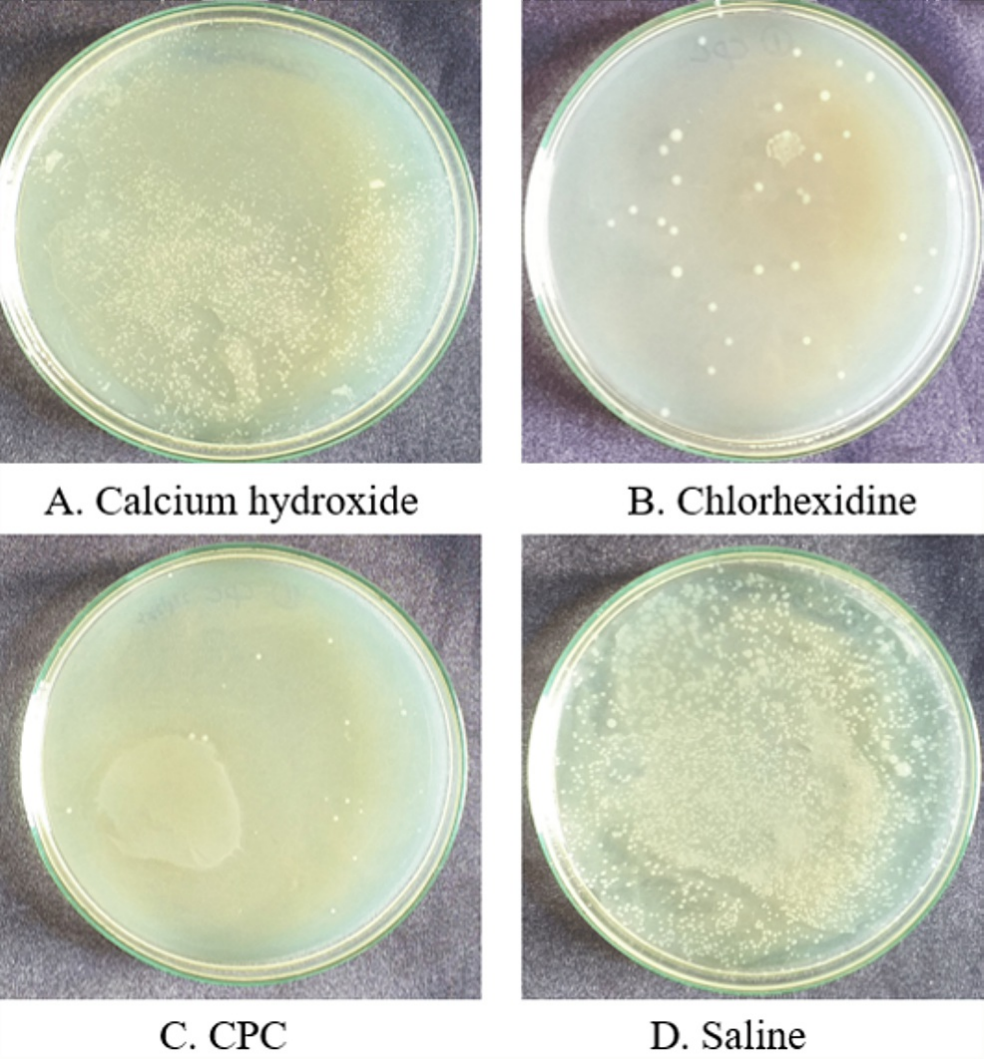
Microbial colony of Enterococcus faecalis of Group B at the 24-hour time interval CPC: cetylpyridinium chloride.

Figure 1 depicts the microbial colony of *Enterococcus faecalis* of Group A at the 24-hour time period and Figure 2 depicts the microbial colony of *Enterococcus faecalis* of Group B at the 24-hour time interval.

**Figure 3:**
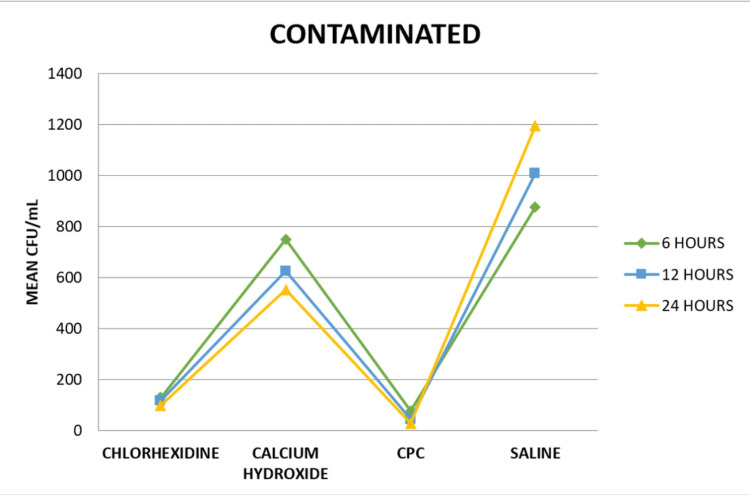
Intra-group comparison of CFU values of the contaminated group at different time intervals CFU: colony-forming unit; CPC: cetylpyridinium chloride.

**Figure 4:**
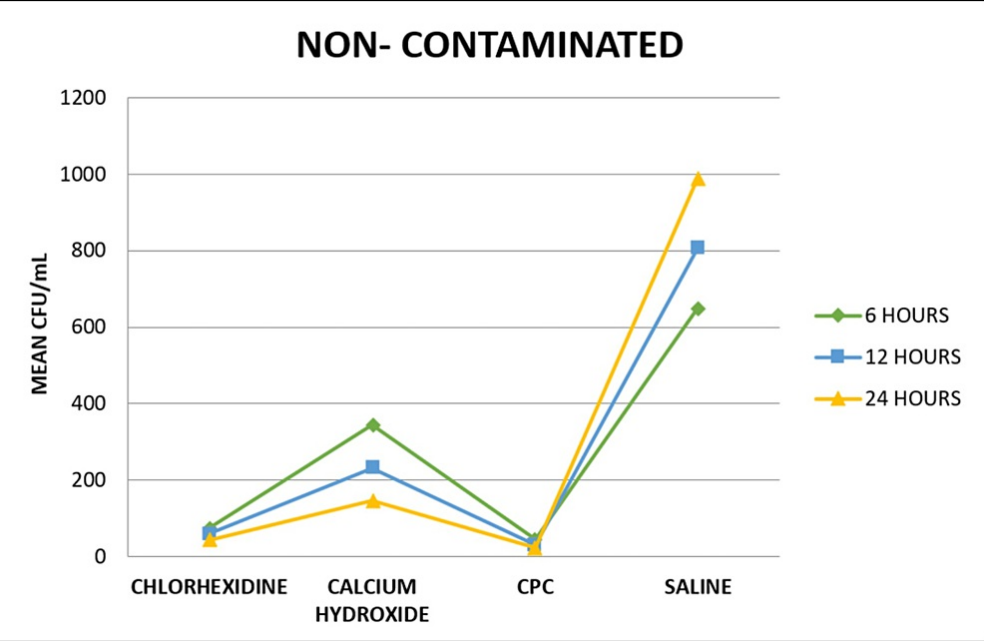
Intra-group comparison of CFU values of the non-contaminated group at different time intervals CFU: colony-forming unit; CPC: cetylpyridinium chloride.

On intra-group comparison of the medicaments at different time intervals, it was seen that there was a statistically significant difference between each medicament at various time intervals in both the contaminated (Group A) and non-contaminated groups (Group B), as seen in Figure 3 and Figure 4, respectively.

On inter-group comparison at six hours, there was a significant difference between the contaminated and non-contaminated group in each medicament. On inter-group comparison at 12 and 24 hours, there was a significant difference between the two groups in CHX and CH solutions, as seen in Table 3.

**Table 3:** Inter-group comparison of CFU values at six, 12, and 24-hour time intervals CFU: colony-forming unit; CPC: cetylpyridinium chloride.

Time period	Solution	Groups	Mean ± SD	Z-value	P-value
6 hours	Chlorhexidine	Contaminated	130.2 ± 25.2	-2.611	0.009
		Non-contaminated	74.2 ± 15.8		
	Calcium hydroxide	Contaminated	748.8 ± 112.2	-2.611	0.009
		Non-contaminated	344.6 ± 81.8		
	CPC	Contaminated	79.2 ± 10.2	-1.984	0.047
		Non-contaminated	44.4 ± 22.9		
	Saline	Contaminated	876.6 ± 82.5	-1.984	0.047
		Non-contaminated	650.6 ± 147.5		
12 hours	Chlorhexidine	Contaminated	118 ± 23.1	-2.611	0.009
		Non-contaminated	61 ± 16.6		
	Calcium hydroxide	Contaminated	626.4 ± 174.4	-2.611	0.009
		Non-contaminated	232 ± 89.2		
	CPC	Contaminated	46 ± 14.7	-1.358	0.175
		Non-contaminated	31 ± 22.9		
	Saline	Contaminated	1007.8 ± 141.4	-1.149	0.251
		Non-contaminated	808.4 ± 197.2		
24 hours	Chlorhexidine	Contaminated	100.4 ± 20	-2.611	0.009
		Non-contaminated	44 ± 11.5		
	Calcium hydroxide	Contaminated	552.6 ± 195.2	-2.611	0.009
		Non-contaminated	145.6 ± 67.1		
	CPC	Contaminated	28 ± 18.5	-0.419	0.675
		Non-contaminated	23.4 ± 20.2		
	Saline	Contaminated	1195.6 ± 191.9	-1.149	0.251
		Non-contaminated	989.8 ± 296.3		

Saline had the highest CFU values while CPC had the lowest CFU values in both the contaminated and non-contaminated groups. In all the conditions, the CFU values of CPC were significantly lowest compared to the other three groups. CFU values of the CH group were significantly high followed by the CHX group. Therefore, the null hypothesis was rejected.

## Discussion

Periapical exudate is most commonly present in the infected root canal of teeth that require endodontic treatment [7]. The antimicrobial effect of various endodontic disinfecting agents is poorer in the root canal than in “neutral” in vitro conditions. Inactivation of the medicaments can occur due to the presence of dentin, enzymes, and serum proteins of periapical exudate [8].

The continuous search for a potent intracanal medicament that can exert its antimicrobial activity despite various inactivators for improved periapical healing is ongoing. Very limited studies are available that simulate the host interactions between the tooth and surrounding tissues [7-10].

*E. faecalis* is an anaerobic gram-positive coccus that normally commences in the human oral cavity. It is the predominant microorganism in secondary infection of root canal-treated teeth, with a prevalence of 24-77% [11].

CH has been widely utilized as an intracanal dressing material for root canal treatment for several years. The bactericidal effect of CH could be attributed due to its high pH of approximately 12.5, wherein the growth and survival of intracanal bacteria can be hindered. CH also denatures the lipopolysaccharide (LPS) present in the dead bacterial cell wall. This medicament is commonly preferred in weeping canals due to its ability to control the inflammatory exudates from the periapical region and has excellent capacity to induce hard tissue formation [12].

In this study, CH exerts a low antibacterial effect against *E. faecalis* in six hours, and a gradual increase in effect is seen in 12 and 24 hours. However, the antibacterial efficacy of CH was lesser than CHX and CPC in both contaminated and non-contaminated groups. Delayed activation of CH can be due to the presence of bovine serum albumin in wound exudate [10].

CHX digluconate, a synthetic cationic bisbiguanide, possesses excellent biocompatibility and wide antimicrobial activity. CHX is a better alternative to CH, which has been shown to be effective against both *E. faecalis* and *Candida albicans*. It has many advantages, including the MMP inhibitory effect, substantivity, and its bactericidal nature in high concentrations. However, it has a few shortcomings like the inability to inactivate LPS from the bacterial cell wall and reduced efficacy on contact with periapical exudates and pulpal remnants, and repeated exposure has led to its resistance against *E. faecalis* [10,13].

In this study, CHX exerts better antimicrobial activity than CH in both contaminated and non-contaminated groups. The reduced efficacy noted might be due to its inactivation in the presence of the exudate [13].

At the end of 12 and 24 hours, the CFU values of CH and CHX between the contaminated and non-contaminated groups were found to be statistically significant. This indicates that there is a significant decline in the antimicrobial activity of CH and CHX against *E. faecalis* in the presence of simulated wound exudate.

CPC is a recently introduced quaternary ammonium compound with unique positively charged surfactant properties. By linking with anionic compounds on the bacterial surface, these surface-active agents with a positively charged part of the molecule have cleansing and antibacterial properties. Thus, the cytoplasmic membrane is disintegrated and an increase in permeability is observed. Later leakage of cell contents, inactivation of enzymes, and protein denaturation occurs, ultimately leading to cell death [2]. Studies showed that CPC incorporated into gutta-percha points and root canal sealers, and as irrigants was effective against *E. faecalis* [3].

CPC had been used as an endodontic irrigant and had shown high efficacy against *E. faecalis*, which was similar to CHX and better than 2.5% sodium hypochlorite (NaOCl) [14]. The minimal bactericidal concentration (MBC) and the minimum inhibitory concentration (MIC) of CPC for *E. faecalis* were found to be 0.0006 and 0.0003%, respectively [15]. CPC has shown low cytotoxicity like CHX but superior biocompatibility.

In this study, there is a constant decline in CFU counts in the six, 12, and 24-hour time intervals with superior antibacterial activity. This may be attributed to various factors. Due to its hydrophilicity, humidity present inside the root canal walls will be helpful in solidifying the gel providing a fluid-tight seal in the apical region, preventing the ingress of periapical fluids into the root canal system, and aiding in superior disinfection [14].

CPC is an effective interim medication compared to CHX as it inhibits LPS binding to toll-like receptors. CPC, unlike CHX, when used as an active therapeutic agent against *E. faecalis*, did not evoke any resistance [14]. A recent study concluded that when CPC was combined with CHX at higher concentrations, a low contact angle was formed with dentin, which indicated a higher penetration depth of the medicament into the dentinal tubules [16]. Newer endodontic sealers were formulated by incorporating CPC with hydroxyethyl methacrylate (HEMA) resin and these sealers have shown sustained anti-bactericidal activity against *E. faecalis* [17].

Unlike other intracanal medicaments that are less efficacious in the presence of wound exudate, the antimicrobial efficacy of CPC was not significantly altered in the presence or absence of the exudate at 12- and 24-hour time intervals. This could be attributed to the fact that CPC has a different mechanism of action against microorganisms rather than local pH alteration. CPC penetrates the bacterial cell wall, disrupts the cell wall integrity, and eventually causes cellular lysis [14].

The antimicrobial efficacy of different intracanal medicaments in the presence or absence of wound exudate was tested in accordance with the methodology adapted from a previous study [10]. Saline was taken as a positive control.

The results obtained from this study are in accordance with previous studies that concluded that the antimicrobial activity of CH and CHX were significantly affected in the presence of bovine serum albumin [8-10].

This is the first in vitro study to examine the effect of contamination of intracanal medicaments with a simulated wound exudate whereas other studies have only considered the effect of bovine serum albumin on the antimicrobial efficacy of medicaments. Novel medicament CPC was tested with the gold standards (CH and CHX). As a preliminary effort to provide standardization among the test samples, this study was performed in vitro.

The limitations of the study are that dentin has a detrimental effect on the antimicrobial efficacy of intracanal medicaments. This was not taken into consideration, as the study was not done using tooth samples. As the study was performed in vitro, ex vivo studies are required to extrapolate the results.

## Conclusions

Despite the advancements in endodontic therapy, the occurrence of secondary re-infections is common due to the persistence of various microorganisms. The inactivation of standard intracanal medicaments occurs in the presence of periapical exudate. Thus, there is a never-ending need for newer and more efficient intracanal medicaments and irrigants with different mechanisms of action for complete microbial elimination from root canals. Within the limitations of the current study, it can be concluded that CPC was the most effective intracanal medicament against *Enterococcus faecalis* than CH and CHX at varying time intervals, even in the presence of a periapical exudate. Thus, CPC can be considered an effective intracanal medicament for root canal disinfection.
